# Supplementary material for: Determinants of photoplethysmography signal quality at the wrist
Source: PLOS Digit Health. 2025 Jun 27;4(6):e0000585. doi: 10.1371/journal.pdig.0000585 (PMC12204546; doi:10.1371/journal.pdig.0000585)
Supplement: Attachment — Submitted filename: Response to Reviewers Comments, PPG signal quality wrist.pdf [file pdig.0000585.s001.pdf]

Determinants of photoplethysmography signal quality at the wrist

Response to Reviewer Comments

PDIG-D-24-00286

We thank the reviewers for their comments, which have helped improve the paper. Below, we provide a point-by-point response to the comments. In the manuscript, the changes we have made are shown in blue text.

Reviewer #1:

The article outlines a methodology for analyzing the key characteristics that ensure high-quality photoplethysmogram (PPG) signal acquisition. While many aspects related to sensor performance have been extensively studied, the impact of factors such as skin tone, sensor height relative to the heart, and posture remains insufficiently explored. The authors aim to quantify the influence of these variables in their study. The article is well-structured and presented in a clear, accessible format. However, I do have some suggestions to improve the manuscript a bit:

1.The introduction lacks sufficient detail on existing literature in this field. While the authors attempt to contextualize their findings within the broader body of research in the discussions section later, it would be beneficial to present these works earlier in the introduction and revisit them in the discussion for better perspective.

Response: *Thank you for highlighting this. We have added further detail on existing literature in the field in a new paragraph in the Introduction. This includes: background on the issue of PPG signal quality in different types of PPG devices; and strategies for addressing the issue of low-quality PPG signals. We have also added to Table I in the Introduction, which now contains 30 references, providing a comprehensive summary of the existing literature on factors influencing PPG signal quality. We have also presented the most relevant works from the discussion in the third paragraph of the Introduction.*

2. Please provide the equation used to calculatedSNR.

Response: *The equation has now been added to Section II.B.*

3. Regarding the correlation with a template, was the template derived from a clean segment of the signal, or was it averaged over the entire signal record? If the template is also generated from noisy data, the template may also be noisy, leading to concerns on whether correlation with such a template would be an indicator of signal quality. Please discuss this a bit more in the method.

Response: *This is an important point. We have now discussed this further in the methods (Section II.B) as follows:*

"The approach of calculating the template pulse wave from all individual pulse waves, regardless of their quality, was chosen to ensure a quantitative assessment of signal quality could be derived from all input signals regardless of their quality. This approach has been used previously [ref], [ref], although we note that others have proposed only using high-quality pulse waves when calculating the template [ref], [ref]."

As mentioned, we think this approach is most helpful for this particular study, where a quantitative assessment of signal quality is required for all input signals. In contrast, the approach of generating a template from only a clean segment of signal may not provide a quantitative assessment for all input signals. This is because, in the case of a noisy record, there may not be sufficient clean signal within the record from which to calculate a template. In this case, it would not be possible to calculate the signal quality. This would be detrimental to the study because it would mean that: (i) some poor quality records would be excluded from the analysis, introducing selection bias; (ii) the exclusion of some records would compromise the paired nature of the data; and (iii) if some records were excluded for the template-matching calculation, then the results would no longer be comparable between the different signal quality metrics.

4. In the mixed-effects analysis, the process by which subject-specific intercepts (random effects) were obtained is unclear. Please clarify this aspect of the methodology.

Response: *We have now clarified this aspect of the methodology as follows: (i) we have clarified the methodology for estimating the parameter of the linear mixed effects model, stating that we used maximum likelihood estimation; (ii) we have clarified that the reason for including subject-specific intercepts was to account for variability in baseline signal quality between subjects; (iii) we have clarified the random effects structure, stating that only random intercepts were used without random slopes; (iv) we have provided examples of the effects that we are (or are not) accounting for to help illustrate the rationale behind the methodology. The updated portions of section Section II.C now read as follows:*

"Linear mixed effects modelling was used to investigate the impact of various factors on PPG signal quality [ref]. The fitlme function in Matlab (R2022a, Mathworks, Natick, USA) was used, with maximum likelihood estimation used to estimate model parameters. … In both models, we included subject-specific intercepts as random effects to model variability in baseline signal quality between subjects which was not explained by the participant characteristics (e.g. variability due to subject-specific differences in blood flow at the wrist). It was possible to account for subject-specific differences at baseline because of the repeated measures per subject. However, we did not include subject-specific random slopes because the number of

repeated measures per subject was considered too small to account for this (e.g. to account for differences between subjects in how a fixed effect such as posture influenced signal quality).”

I look forward to reading your article after the revisions.

Reviewer #2:

The authors present an extensive analysis on factors influencing wrist PPG signal quality. The work uses three signal quality measures to evaluate the influence of posture, sensor height and participant characteristics on a large freely available dataset. Overall, the work is well written and important fundamental research.

I have some minor comments and questions for the authors:

- In Figure 1 no unit on the y axis is shown. As in Figure 2, the unit should be set to a.u..

Response: *Thank you. We have now added the units.*

- In line 143 it is stated that the Wilcoxon tests were performed at a significance level of 5%. In figure 5 and 6, statistically significant differences are reported, when $p<0.001$, indicating a significance level of 0.1%. The authors should unify this.

Response: *Tests were indeed performed at a significant level of 5%. Our mention of $p<0.001$ in Figures 5 and 6 was ambiguous. We have now removed the mention of $p<0.001$ in Figures 5 and 6.*

- Also, in Figure 5 and 6, there are some indicators of significance in brackets, others are not. Does this have any meaning? If so, the authors should state this in the caption. Also, in Figure 6, the statement regarding significant differences in the lower row on the left needs more space. Generally, the authors should consider using significance bars in the plot for easier to grasp significant differences.

Response: *Thank you for highlighting that some indicators of significance were in brackets whilst others were not. This was not intentional. As suggested, we have now removed the indicators of significance in the brackets, and have replaced them with significance bars. This avoided the issue with there not being enough space for the statement regarding significant differences.*

- If I understand correctly, each group in the Wilcoxon test comparisons contains multiple observations of the same subjects. If this is the case, then in such a comparison, the subjects should be regarded as a random effect, because the samples of a group are not independent anymore.

Response: *Thank you for highlighting this. You are correct, there were multiple observations of the same subjects. Specifically, in the Oscillometric protocol, which was the subset used for the Wilcoxon test comparisons, there were two observations per subject for the supine posture, and one observation per subject for the other postures (see Table II for details). We have now changed the statistical methods, to use a linear mixed effects model instead of the Wilcoxon test. Posture (or sensor height) was included as a fixed effect, and the subject was included as a random effect. This is now described in the manuscript (Section II.C.) as follows:*

"Linear mixed effects modelling was used to investigate the impact of various factors on PPG signal quality [ref]. The fitlme function in Matlab (R2022a, Mathworks, Natick, USA) was used, with maximum likelihood estimation used to estimate model parameters. First, the impact of posture and sensor height on PPG signal quality was assessed using direct comparisons on the Oscillometric Subset. Since each participant in this subset contributed the same number of recordings at each posture and sensor height, it was possible to perform direct comparisons of PPG signal quality between different postures and sensor heights. To do so, a mixed effects model was fitted for each signal quality metric, with posture (or sensor height) specified as a fixed effect, and subject-specific intercepts included as random effects. These models were used to test for significant differences between different postures and sensor heights at the 5% significance level, using the p-value from the model's fixed-effects coefficient estimates."

- Also in the conducted statistical analysis (Figure 5 and 6), the authors compare all groups against one another. Therefore, the analysis should be corrected for multiple comparisons.

Response: *Thank you. We have now implemented a Holm-Sidak correction for multiple comparisons, to ensure that the probability of a type I error is fixed at 5%. This is now described in the manuscript (Section II.C) as: "A total of 30 tests were performed (10 tests for each of the 3 signal quality metrics). Therefore, a Holm-Sidak correction was used to correct for multiple comparisons [ref], [ref], as implemented in [ref]." All of the significant differences remained significant after implementing this correction.*

- Two minor spelling mistakes caught my eye:
  - o In line 56 'acqusition' should be changed to 'acquisition'.
  - o In line 249 'event' should be changed to 'even'.

Response: *Thank you. These have now been corrected.*
